# Supplementary material for: Combinatorial bZIP dimers display complex DNA-binding specificity landscapes
Source: eLife. 2017 Feb 10;6:e19272. doi: 10.7554/eLife.19272 (PMC5349851; doi:10.7554/eLife.19272)
Supplement: Supplementary file 2. — DOI: http://dx.doi.org/10.7554/eLife.19272.023 [file elife-19272-supp2.pdf]

This is Supplementary data for Combinatorial bZIP dimers define complex DNA-binding specificity landscapes by José A. Rodríguez-Martínez, Aaron W. Reinke, Devesh Bhimsaria, Amy E. Keating, Aseem Z. Ansari 2017.

Supplementary file 2. MEME motifs and Sequence Specificity and Energy Landscapes (SEL) for human bZIP homodimers and heterodimers. **column 1**, bZIP dimer and dissociation constant of bZIP dimerization in the absence of DNA, from Reinke et al. (Science, 2013); **column 2**, Representative MEME motifs derived using the top 1,000 12-mer sequences ranked by CSI intensity; **column 3**, SEL displaying CSI intensities of 12-mer sequences in the 0-, 1-, 2- mismatch rings. Seed motifs used to draw SELs are shown on top; **column 4**, Box plot of CSI intensities corresponding to the first three rings (0-, 1-, 2-mismatch rings) of the SEL. bZIP heterodimer motifs were classified as Conjoined (C), Variably-spaced (V), or Emergent (E) based on the DNA binding preferences of the composing bZIPs and a notation of the category included between columns 2 and 3. Multiple MEME motifs were identified for some homodimers and heterodimers. In such cases, motifs are bracketed in column 1.

Homodimer (PPI, Kd)	MEME motif	Sequence Specificity & Energy Landscape (SEL)	Box plot for SEL rings
ATF2 (32 nM)	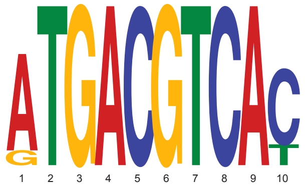	ATGACGTCAT 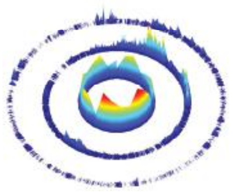	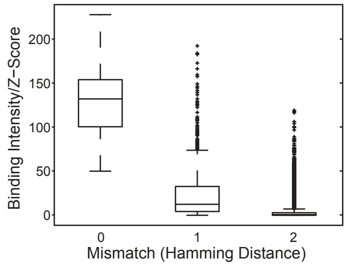
ATF3 (218 nM)	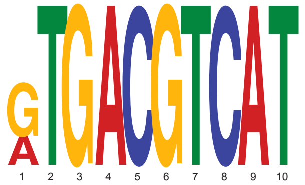	ATGACGTCAT 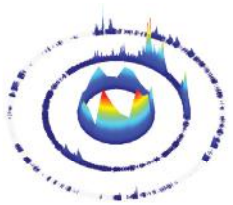	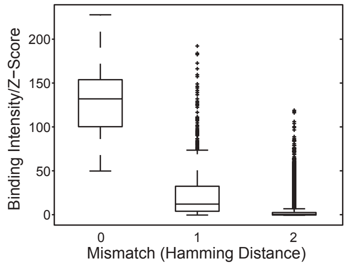
ATF4 (412 nM)	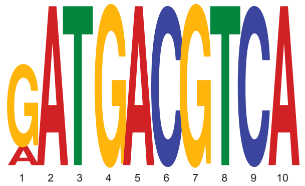	ATGACGTCAT 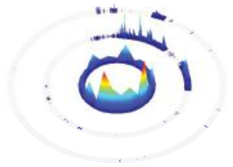	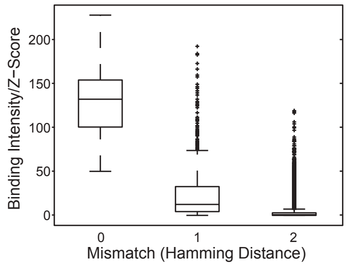
ATF6 (27 nM)	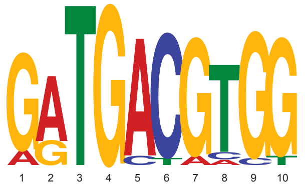	ATGACGTGGC 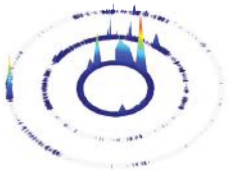	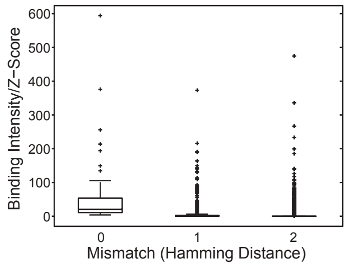
ATF6 (27 nM)	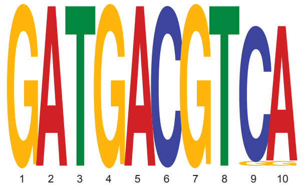	ATGACGTCAT 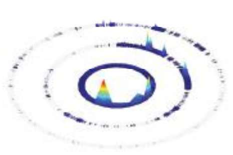	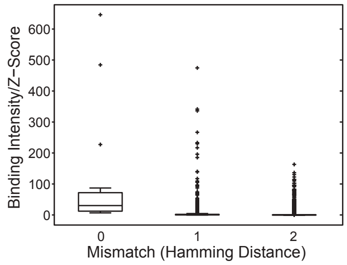

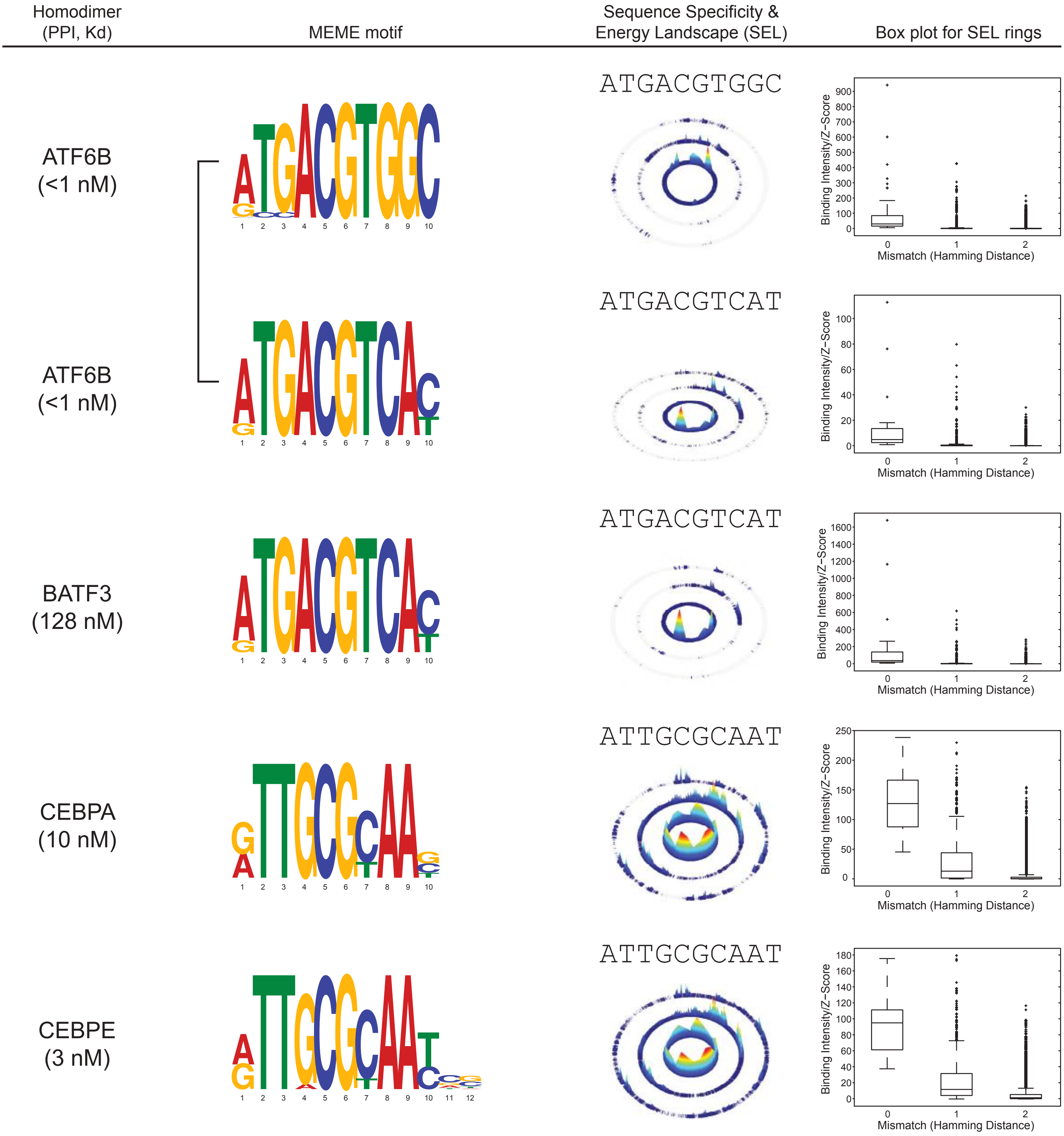
Homodimer
(PPI, Kd)
MEME motif
Sequence Specificity &
Energy Landscape (SEL)
Box plot for SEL rings
ATF6B
(<1 nM)
ATGACGTGGC
ATF6B
(<1 nM)
ATGACGTCAT
BATF3
(128 nM)
ATGACGTCAT
CEBPA
(10 nM)
ATTGCGCAAT
CEBPE
(3 nM)
ATTGCGCAAT
Binding Intensity/Z-Score
Mismatch (Hamming Distance)

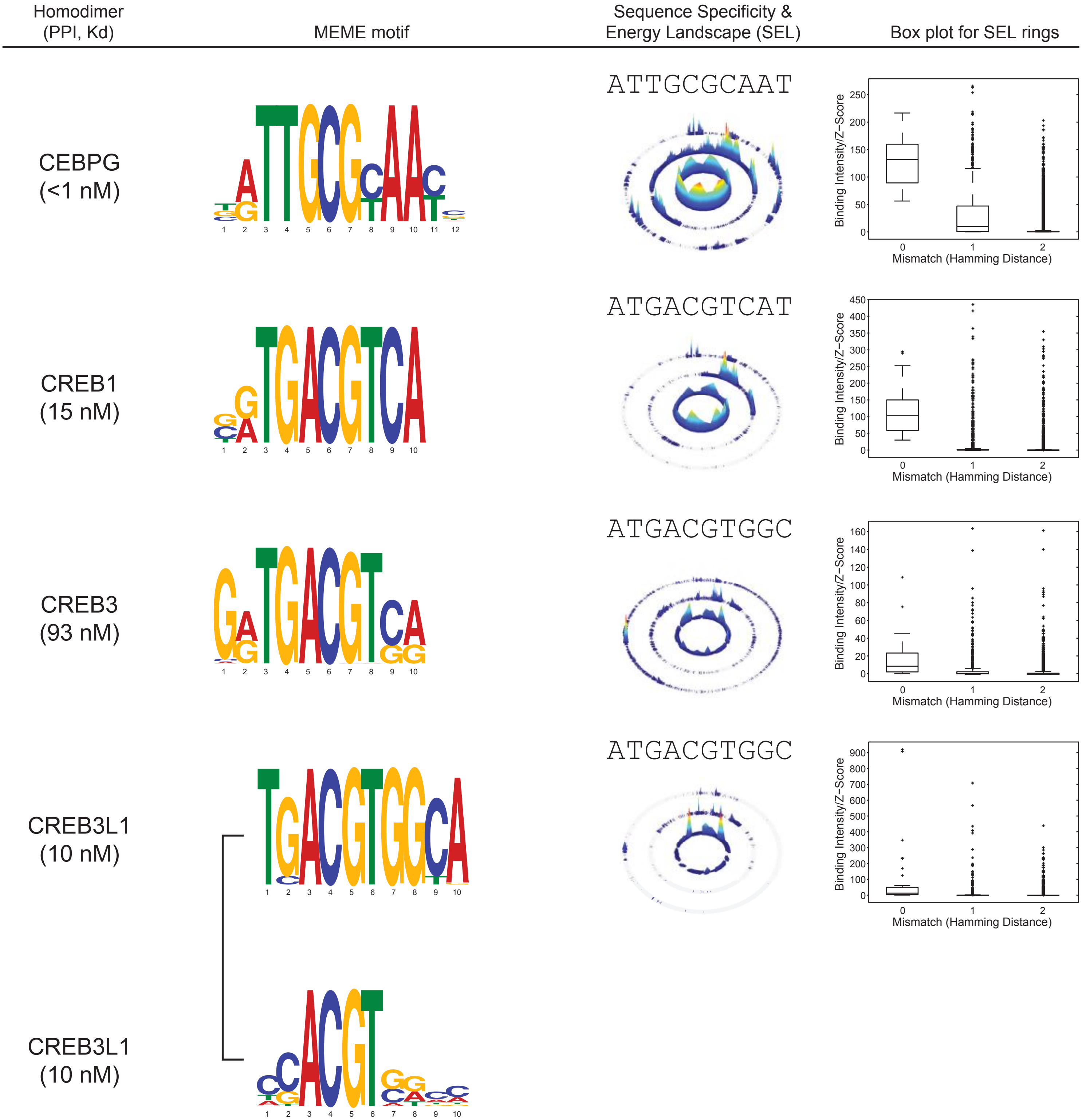

Homodimer
(PPI, Kd)
MEME motif
Sequence Specificity &
Energy Landscape (SEL)
Box plot for SEL rings
CEBPG
(<1 nM)
ATTGCGCAAT
CREB1
(15 nM)
ATGACGTCAT
CREB3
(93 nM)
ATGACGTGGC
CREB3L1
(10 nM)
ATGACGTGGC
CREB3L1
(10 nM)
Binding Intensity/Z−Score
Mismatch (Hamming Distance)

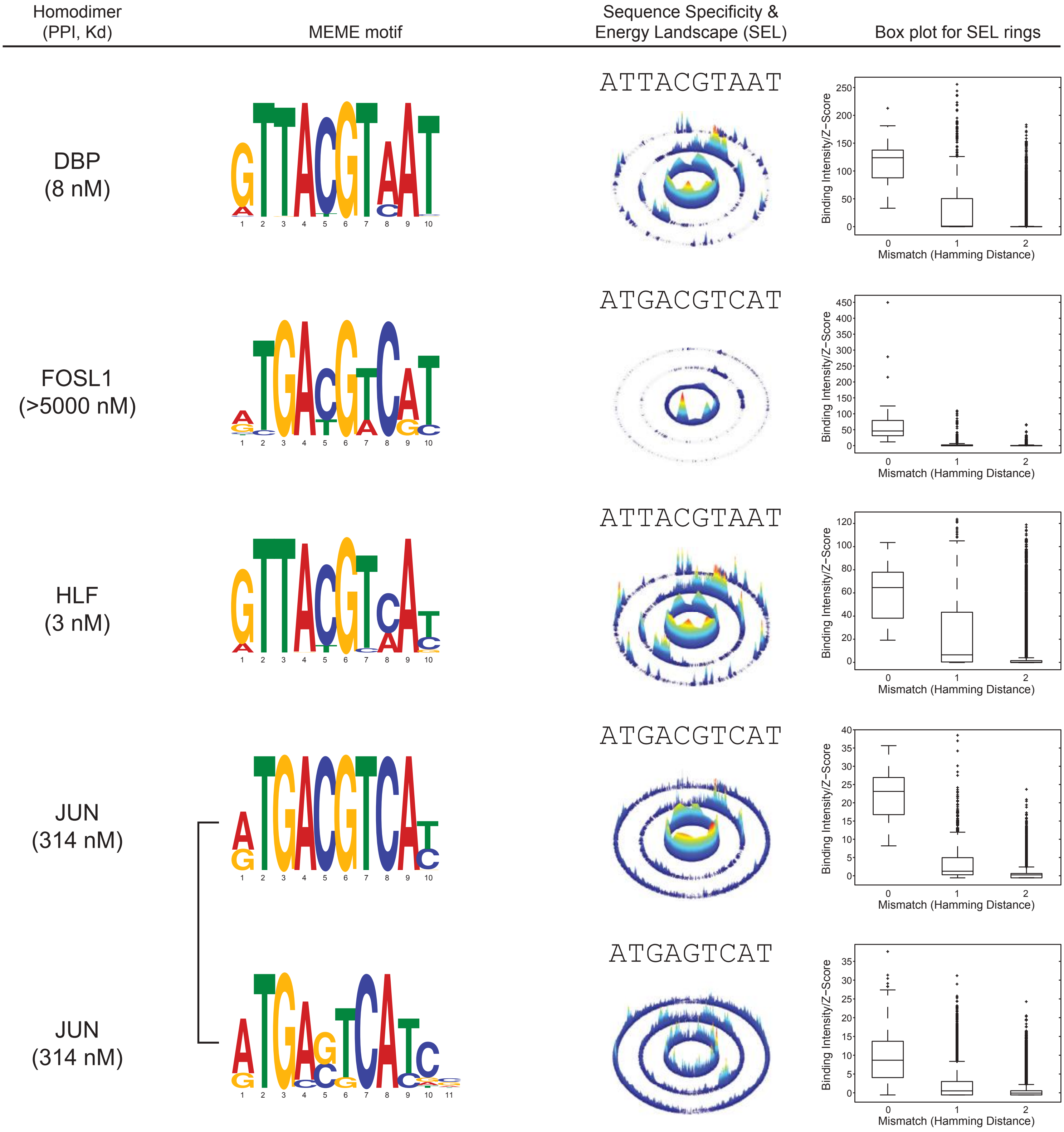

Homodimer
(PPI, Kd)
MEME motif
Sequence Specificity &
Energy Landscape (SEL)
Box plot for SEL rings
DBP
(8 nM)
ATTACGTAAT
FOSL1
(>5000 nM)
ATGACGTCAT
HLF
(3 nM)
ATTACGTAAT
JUN
(314 nM)
ATGACGTCAT
JUN
(314 nM)
ATGAGTCAT
Binding Intensity/Z-Score
Mismatch (Hamming Distance)

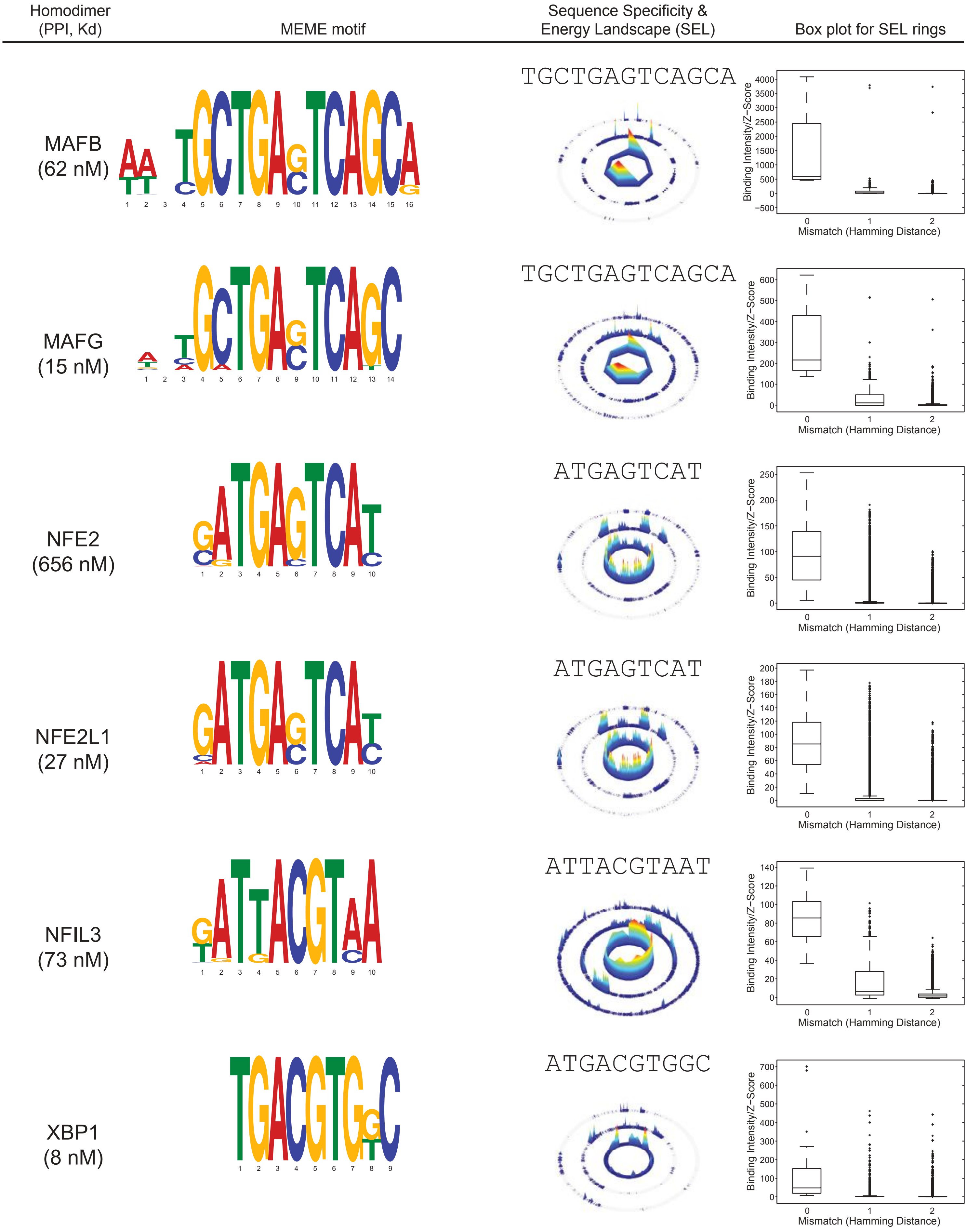

Homodimer
(PPI, Kd)
MEME motif
Sequence Specificity &
Energy Landscape (SEL)
Box plot for SEL rings
MAFB
(62 nM)
TGCTGAGTCAGCA
MAFG
(15 nM)
TGCTGAGTCAGCA
NFE2
(656 nM)
ATGAGTCAT
NFE2L1
(27 nM)
ATGAGTCAT
NFIL3
(73 nM)
ATTACGTAAT
XBP1
(8 nM)
ATGACGTGGC
Binding Intensity/Z-Score
Mismatch (Hamming Distance)

Heterodimer (PPI, Kd)	MEME motif and classification as (i) conjoined half-sites (C), (ii) conjoined half-site with a variable spacer (V), or as (iii) emergent (E) based on homodimer specificity.		Sequence Specificity & Energy Landscape (SEL)	Box plot for SEL rings
ATF3•CEBPA (13 nM)	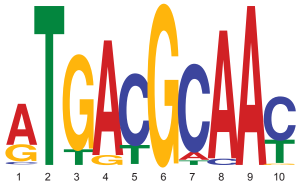	C	ATGACGCAAT 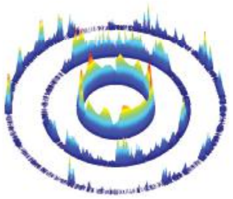	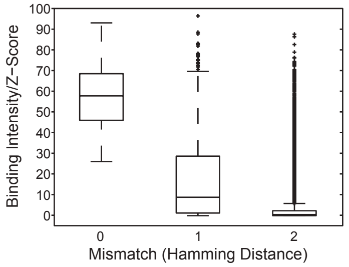
ATF3•CEBPG (<1 nM)	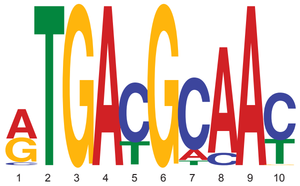	C	ATGACGCAAT 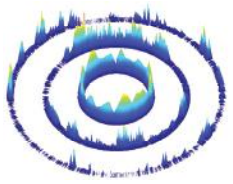	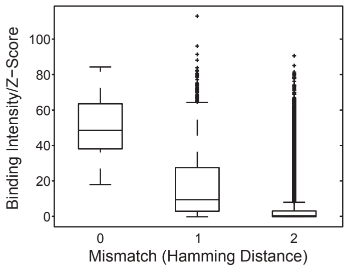
ATF4•CEBPA (<1 nM)	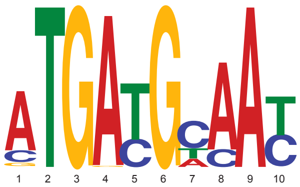	C	ATGACGCAAT 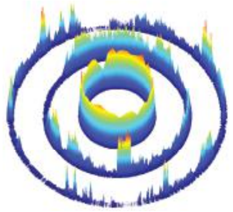	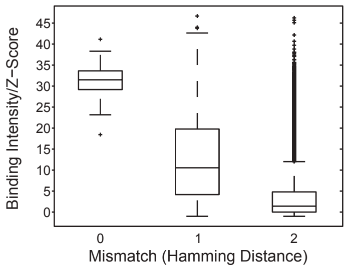
ATF4•CEBPG (<1 nM)	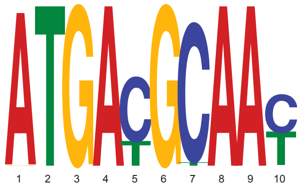	C	ATGACGCAAT 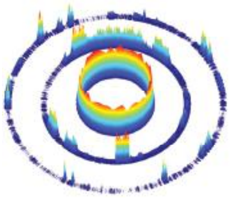	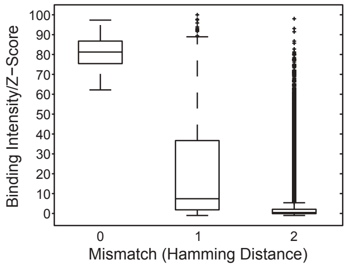
ATF5•CEBPA (54 nM)	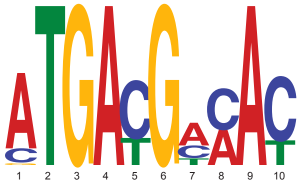	C	ATGACGCAAT 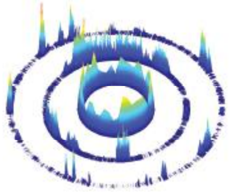	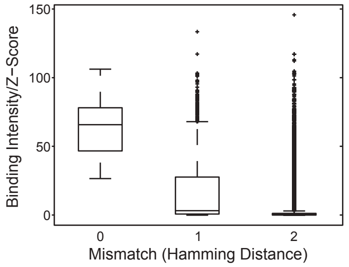
ATF5•CEBPE (28 nM)	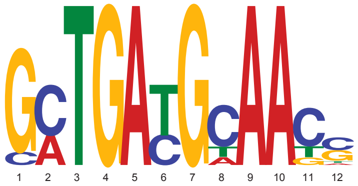	C	CTGATGCAAC 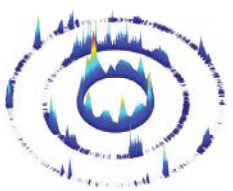	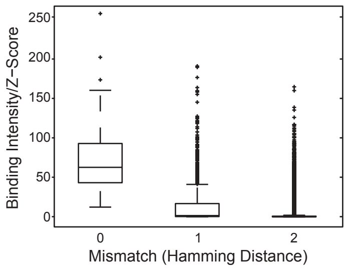

Heterodimer (PPI, Kd)	MEME motif and classification as (i) conjoined half-sites (C), (ii) conjoined half-site with a variable spacer (V), or as (iii) emergent (E) based on homodimer specificity.		SeqEence Specificity & Energy Landscape (SEL)	Box plot for SEL rings
ATF5•CEBPG (<1 nM)	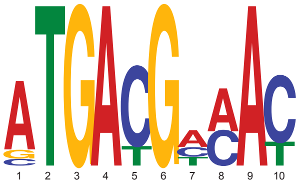	C	ATGACGCAAT 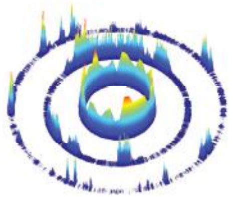	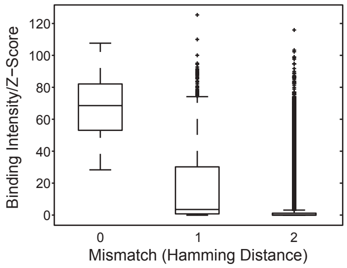
ATF5•ATF6B (>5000 nM)	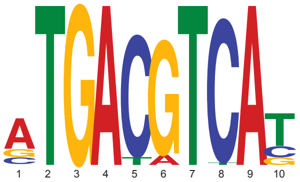	C	ATGACGTCAT 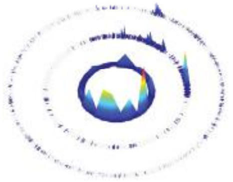	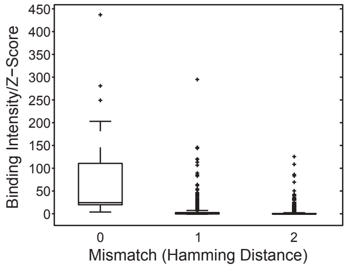
ATF5•BATF (187 nM)	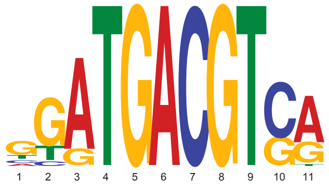	C	ATGACGTCAT 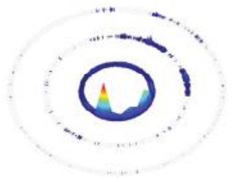	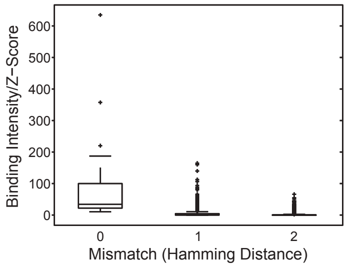
ATF5•BATF2 (>5000 nM)	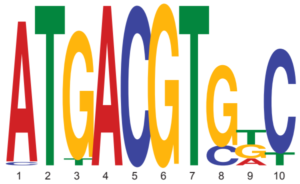	E	ATGACGTGGC 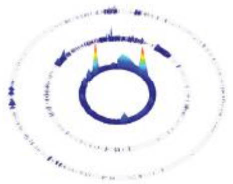	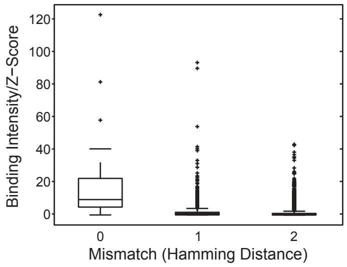
ATF5•BATF3 (385 nM)	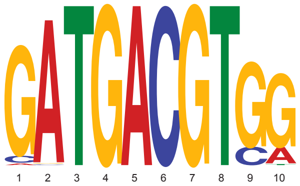	E	ATGACGTGGC 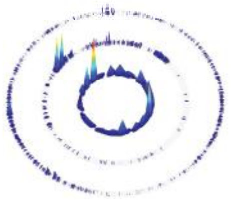	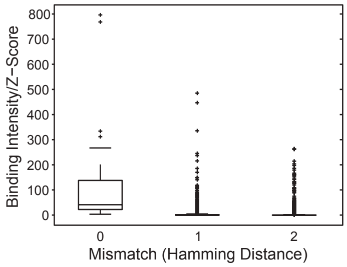
ATF5•CREBZF (1541 nM)	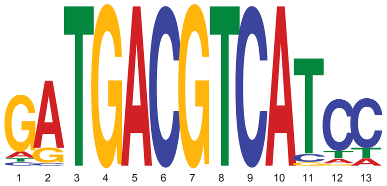	C	ATGACGTCAT 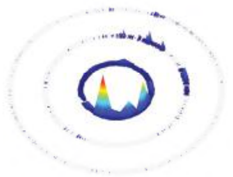	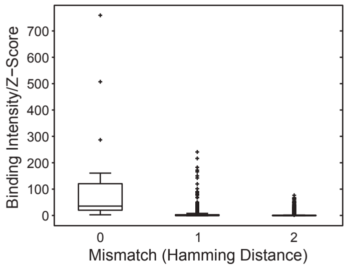

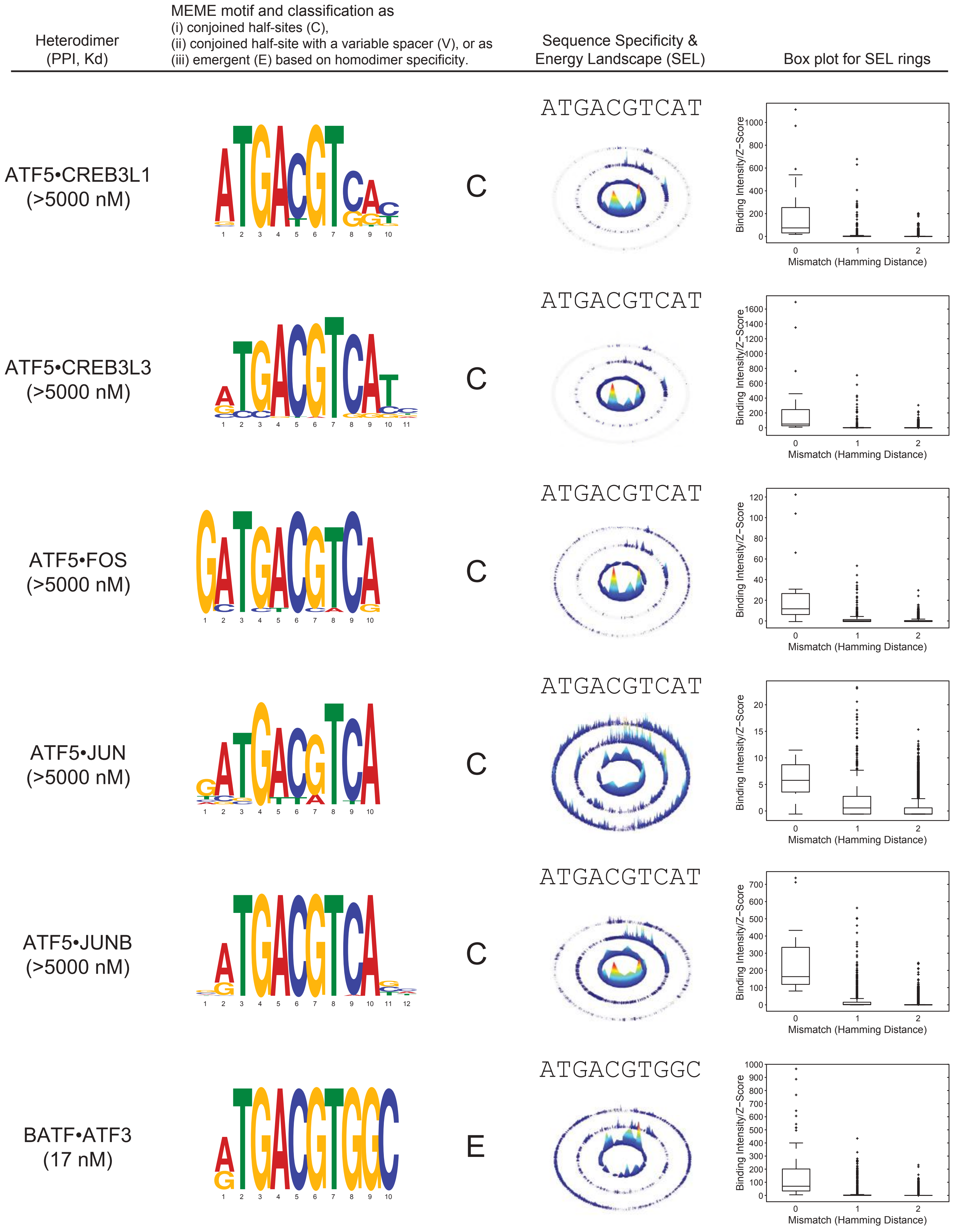
Heterodimer (PPI, Kd)
MEME motif and classification as
(i) conjoined half-sites (C),
(ii) conjoined half-site with a variable spacer (V), or as
(iii) emergent (E) based on homodimer specificity.
Sequence Specificity & Energy Landscape (SEL)
Box plot for SEL rings
ATF5•CREB3L1 (>5000 nM)
C
ATGACGTCAT
ATF5•CREB3L3 (>5000 nM)
C
ATGACGTCAT
ATF5•FOS (>5000 nM)
C
ATGACGTCAT
ATF5•JUN (>5000 nM)
C
ATGACGTCAT
ATF5•JUNB (>5000 nM)
C
ATGACGTCAT
BATF•ATF3 (17 nM)
E
ATGACGTGGC
Binding Intensity/Z-Score
Mismatch (Hamming Distance)

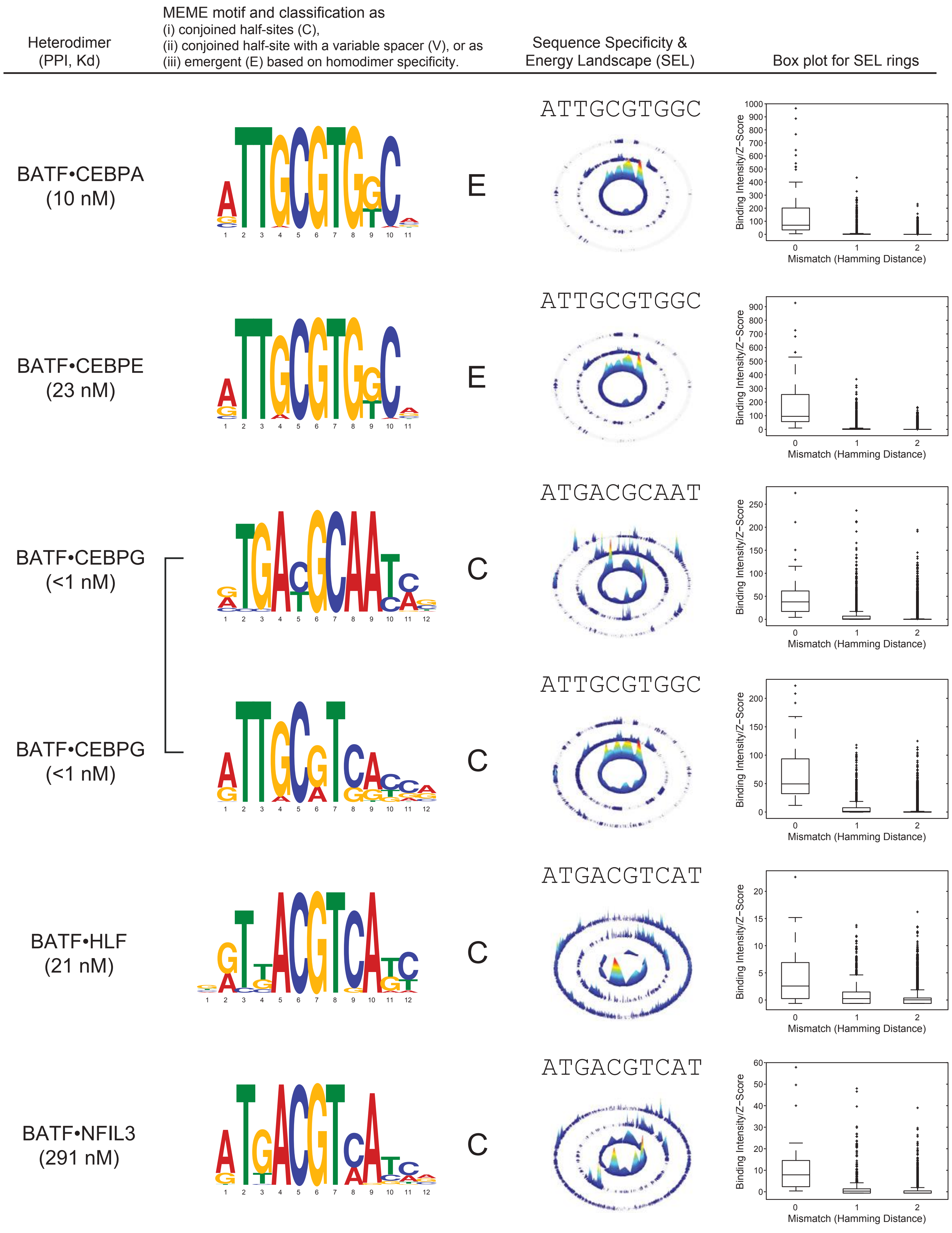

Heterodimer
(PPI, Kd)
MEME motif and classification as
(i) conjoined half-sites (C),
(ii) conjoined half-site with a variable spacer (V), or as
(iii) emergent (E) based on homodimer specificity.
Sequence Specificity &
Energy Landscape (SEL)
Box plot for SEL rings
BATF•CEBPA
(10 nM)
E
ATTGCGTGGC
BATF•CEBPE
(23 nM)
E
ATTGCGTGGC
BATF•CEBPG
(<1 nM)
C
ATGACGCAAT
BATF•CEBPG
(<1 nM)
C
ATTGCGTGGC
BATF•HLF
(21 nM)
C
ATGACGTCAT
BATF•NFIL3
(291 nM)
C
ATGACGTCAT
Binding Intensity/Z-Score
Mismatch (Hamming Distance)

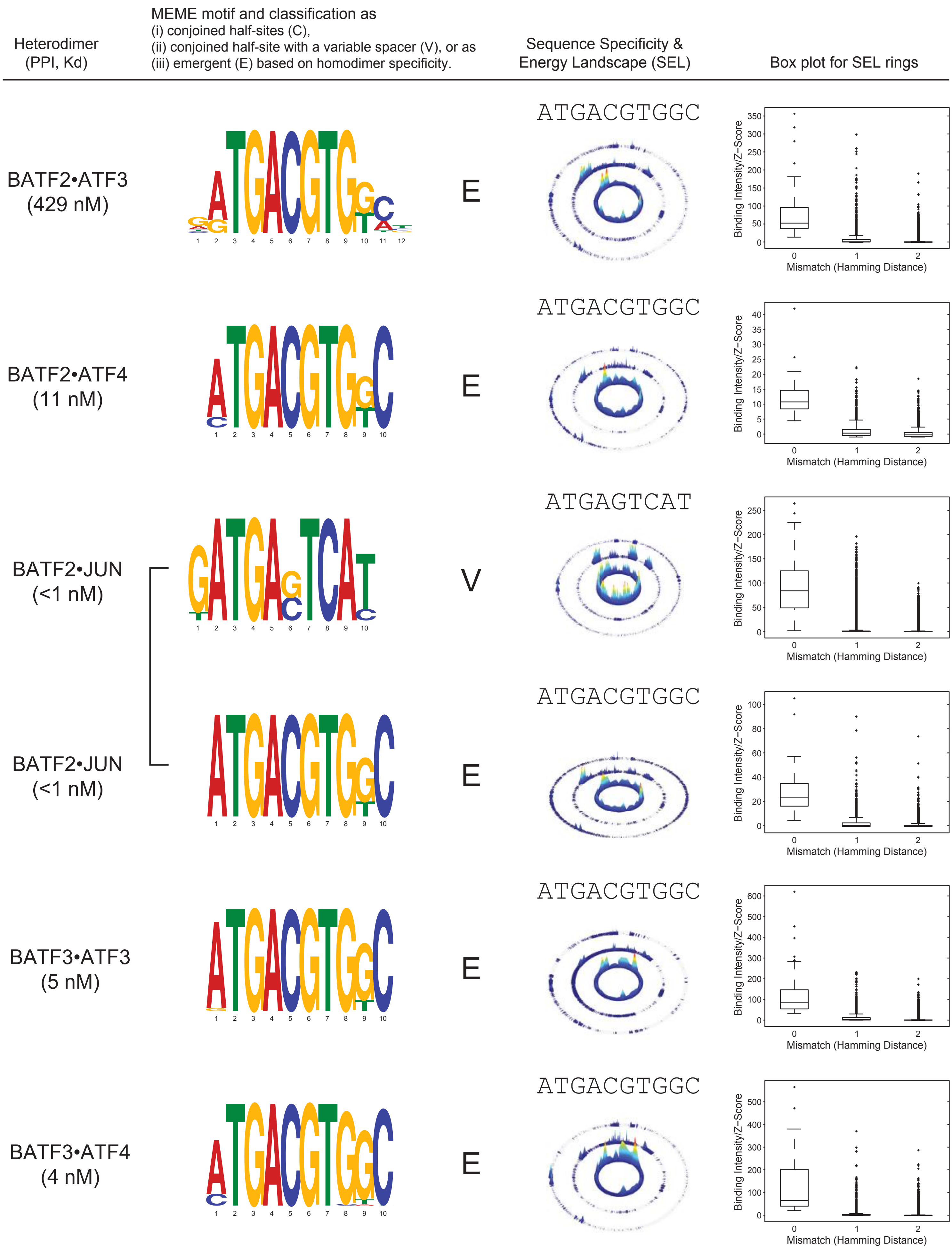
Heterodimer (PPI, Kd)
MEME motif and classification as
(i) conjoined half-sites (C),
(ii) conjoined half-site with a variable spacer (V), or as
(iii) emergent (E) based on homodimer specificity.
Sequence Specificity & Energy Landscape (SEL)
Box plot for SEL rings
BATF2•ATF3 (429 nM)
E
ATGACGTGGC
BATF2•ATF4 (11 nM)
E
ATGACGTGGC
BATF2•JUN (<1 nM)
V
ATGAGTCAT
BATF2•JUN (<1 nM)
E
ATGACGTGGC
BATF3•ATF3 (5 nM)
E
ATGACGTGGC
BATF3•ATF4 (4 nM)
E
ATGACGTGGC
Binding Intensity/Z-Score
Mismatch (Hamming Distance)

Heterodimer (PPI, Kd)	MEME motif and classification as (i) conjoined half-sites (C), (ii) conjoined half-site with a variable spacer (V), or as (iii) emergent (E) based on homodimer specificity.		Sequence Specificity & Energy Landscape (SEL)	Box plot for SEL rings
BATF3•CEBPA (5 nM)	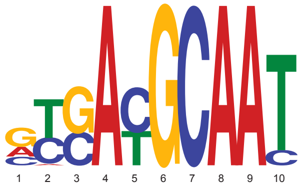	C	ATGACGCAAT 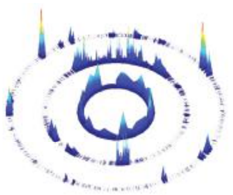	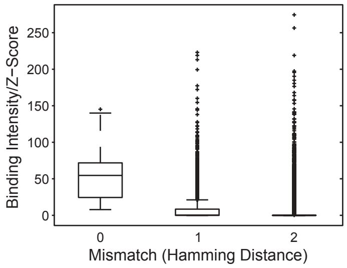
BATF3•CEBPG (2 nM)	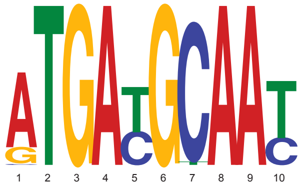	C	ATGACGCAAT 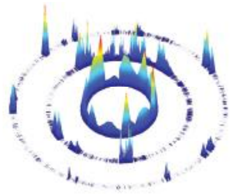	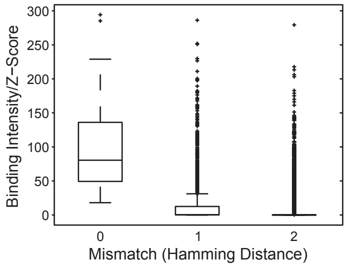
BATF3•NFIL3 (150 nM)	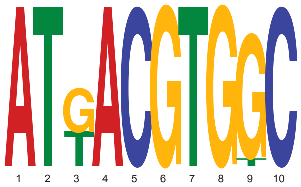	E	ATGACGTGGC 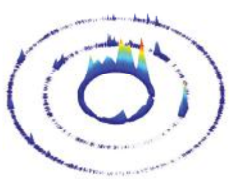	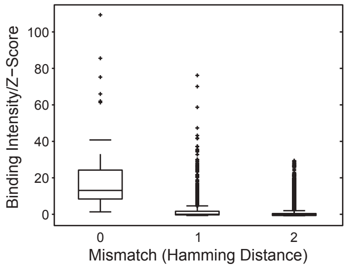
DDIT3•CEBPG (<1 nM)	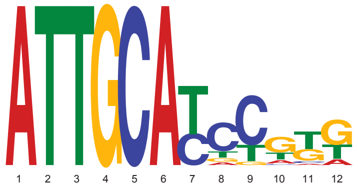	E	ATTGCA 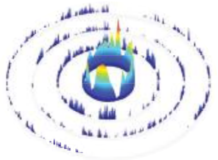	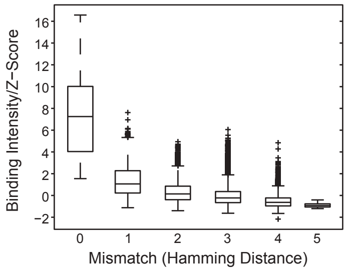
FOS•ATF2 (5 nM)	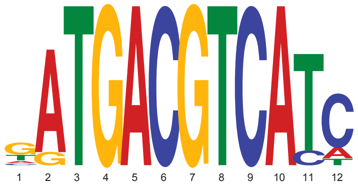	C	ATGACGTCAT 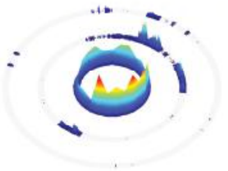	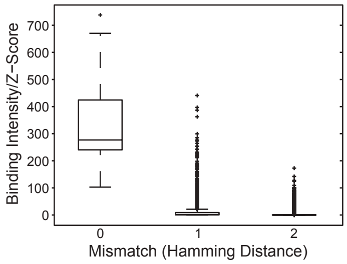
FOS•ATF3 (185 nM)	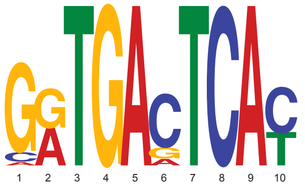	V	ATGAGTCAT 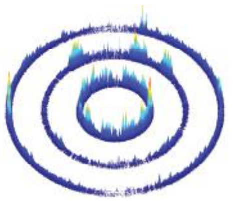	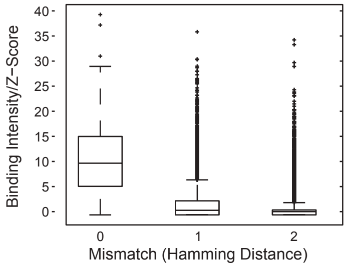

Heterodimer (PPI, Kd)	MEME motif and classification as (i) conjoined half-sites (C), (ii) conjoined half-site with a variable spacer (V), or as (iii) emergent (E) based on homodimer specificity.		Sequence Specificity & Energy Landscape (SEL)	Box plot for SEL rings
FOS•ATF4 (4 nM)	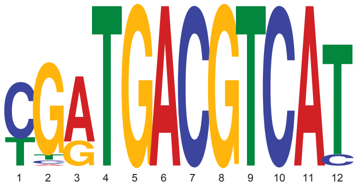	C	ATGACGTCAT 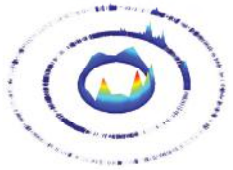	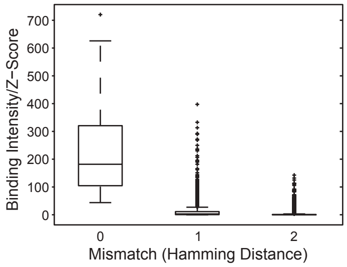
FOS•BATF (3593 nM)	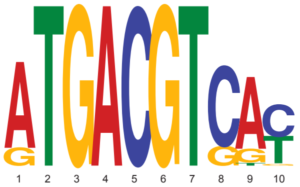	C	ATGACGTCAT 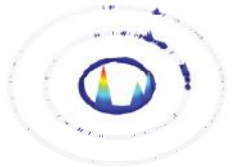	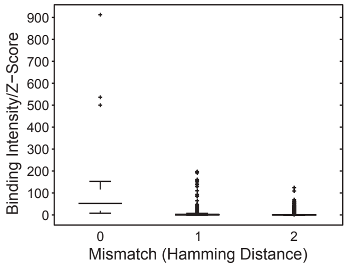
FOS•BATF2 (>5000 nM)	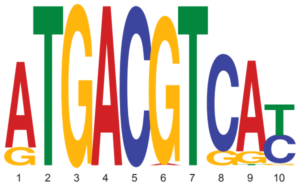	C	ATGACGTCAT 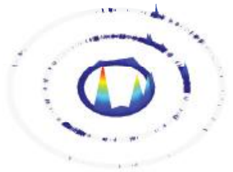	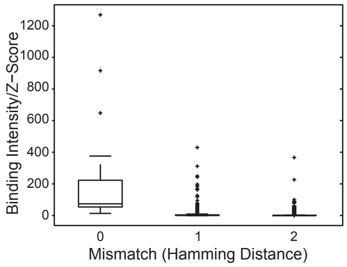
FOS•BATF3 (481nM)	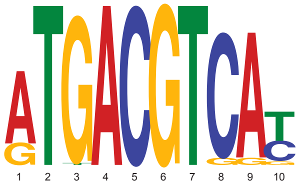	C	ATGACGTCAT 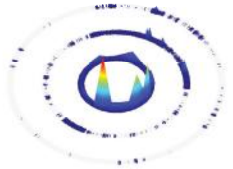	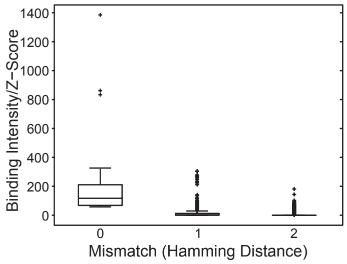
FOS•CEBPE (239nM)	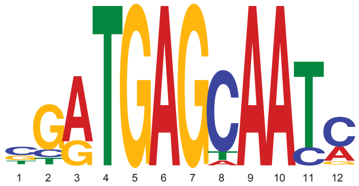	V	ATGAGCAAT 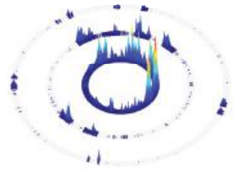	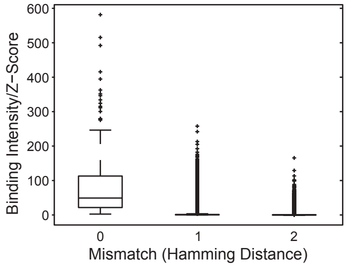
FOS•CEBPG (26nM)	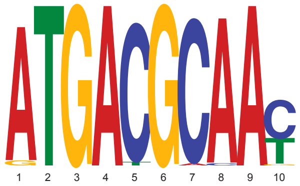	C	ATGACGCAAT 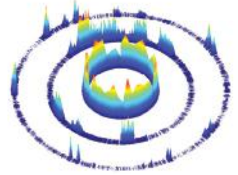	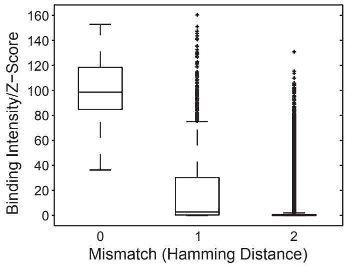

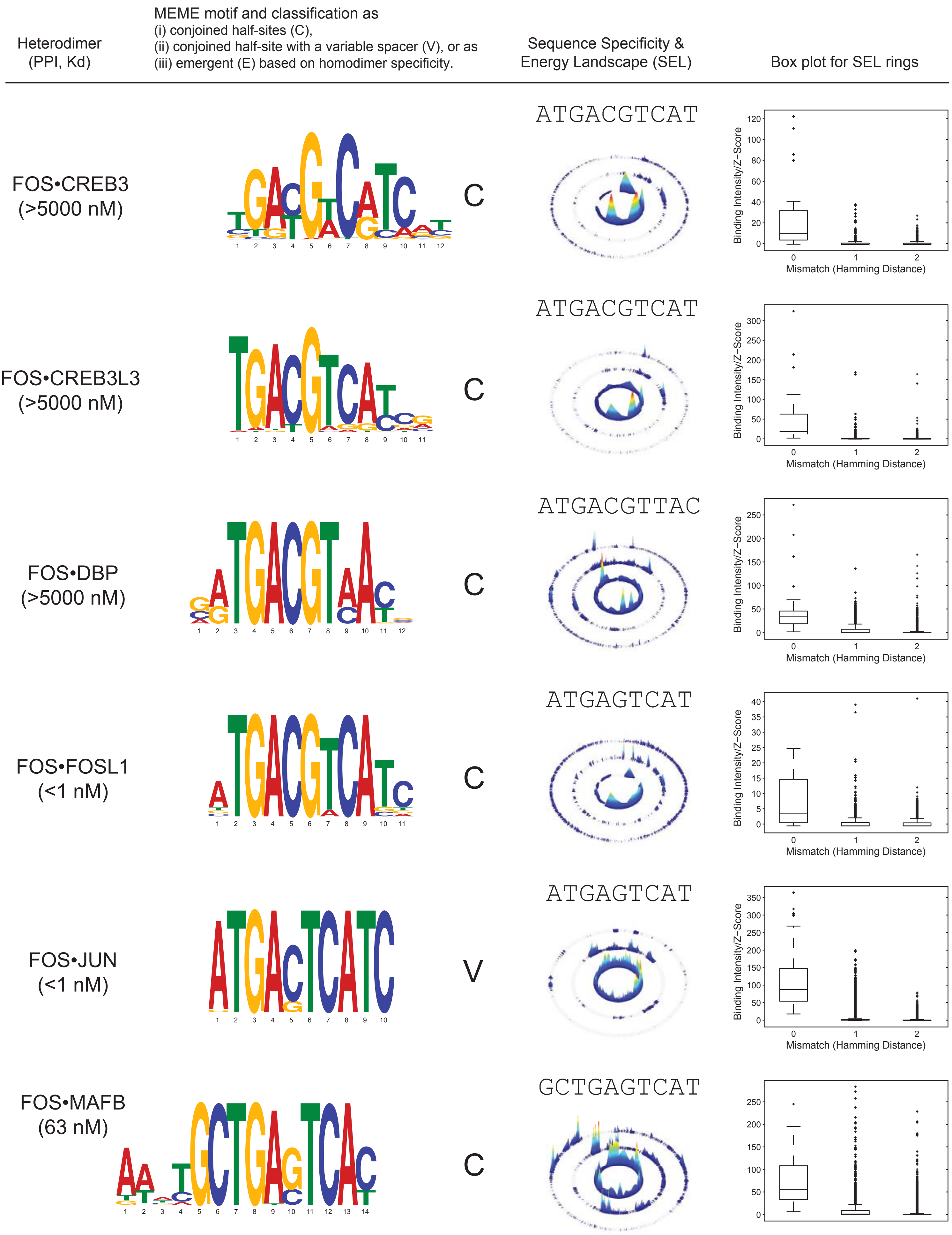
Heterodimer
(PPI, Kd)
MEME motif and classification as
(i) conjoined half-sites (C),
(ii) conjoined half-site with a variable spacer (V), or as
(iii) emergent (E) based on homodimer specificity.
Sequence Specificity &
Energy Landscape (SEL)
Box plot for SEL rings
FOS•CREB3
(>5000 nM)
C
ATGACGTCAT
FOS•CREB3L3
(>5000 nM)
C
ATGACGTCAT
FOS•DBP
(>5000 nM)
C
ATGACGTTAC
FOS•FOSL1
(<1 nM)
C
ATGAGTCAT
FOS•JUN
(<1 nM)
V
ATGAGTCAT
FOS•MAFB
(63 nM)
C
GCTGAGTCAT
Binding Intensity/Z-Score
Mismatch (Hamming Distance)

Heterodimer (PPI, Kd)	MEME motif and classification as (i) conjoined half-sites (C), (ii) conjoined half-site with a variable spacer (V), or as (iii) emergent (E) based on homodimer specificity.		Sequence Specificity & Energy Landscape (SEL)	Box plot for SEL rings

FOS•NFE2 (>5000 nM)

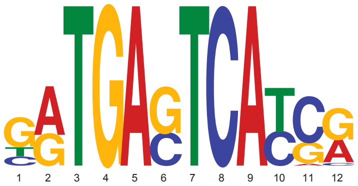

C

ATGAGTCAT

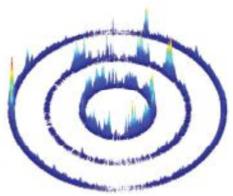

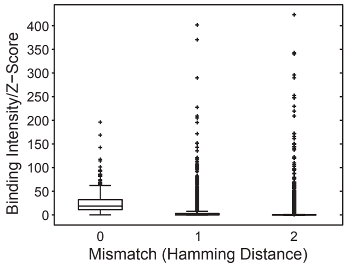

FOS•NFE2L1 (>5000 nM)

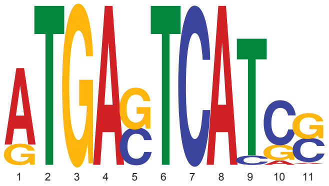

C

ATGAGTCAT

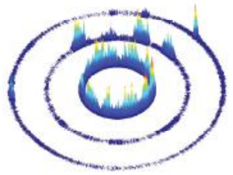

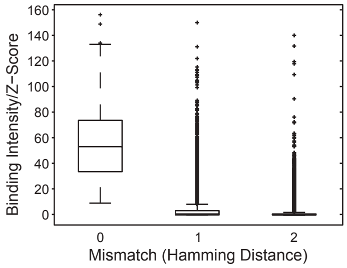

FOSL1•ATF3 (233 nM)

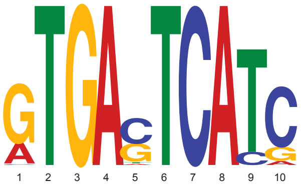

V

ATGAGTCAT

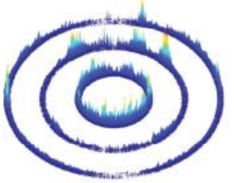

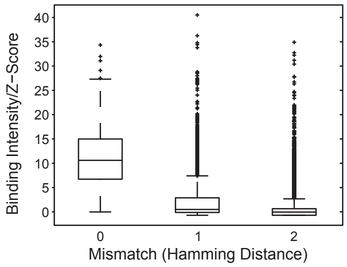

FOSL1•BATF3 (481 nM)

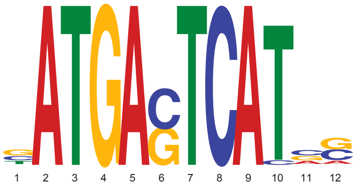

V

ATGAGTCAT

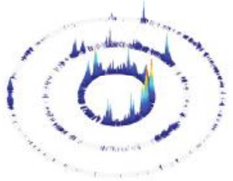

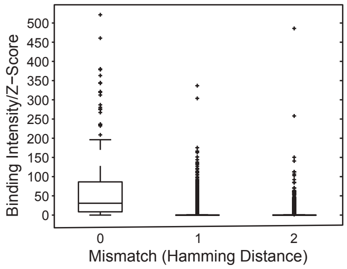

FOSL1•CEBPA (349 nM)

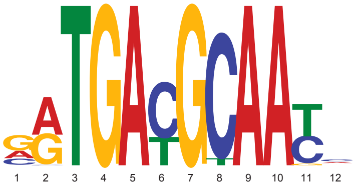

C

ATGACGCAAT

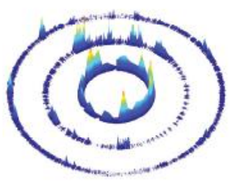

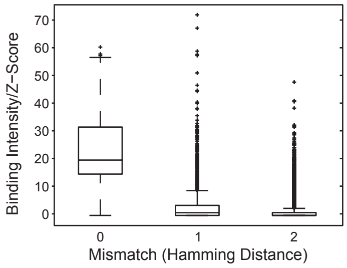

FOSL1•CEBPE (266 nM)

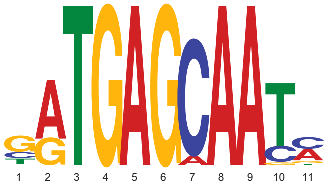

V

ATGAGCAAT

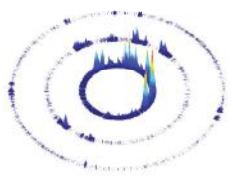

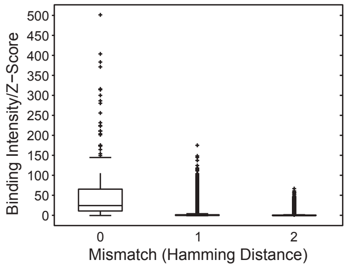

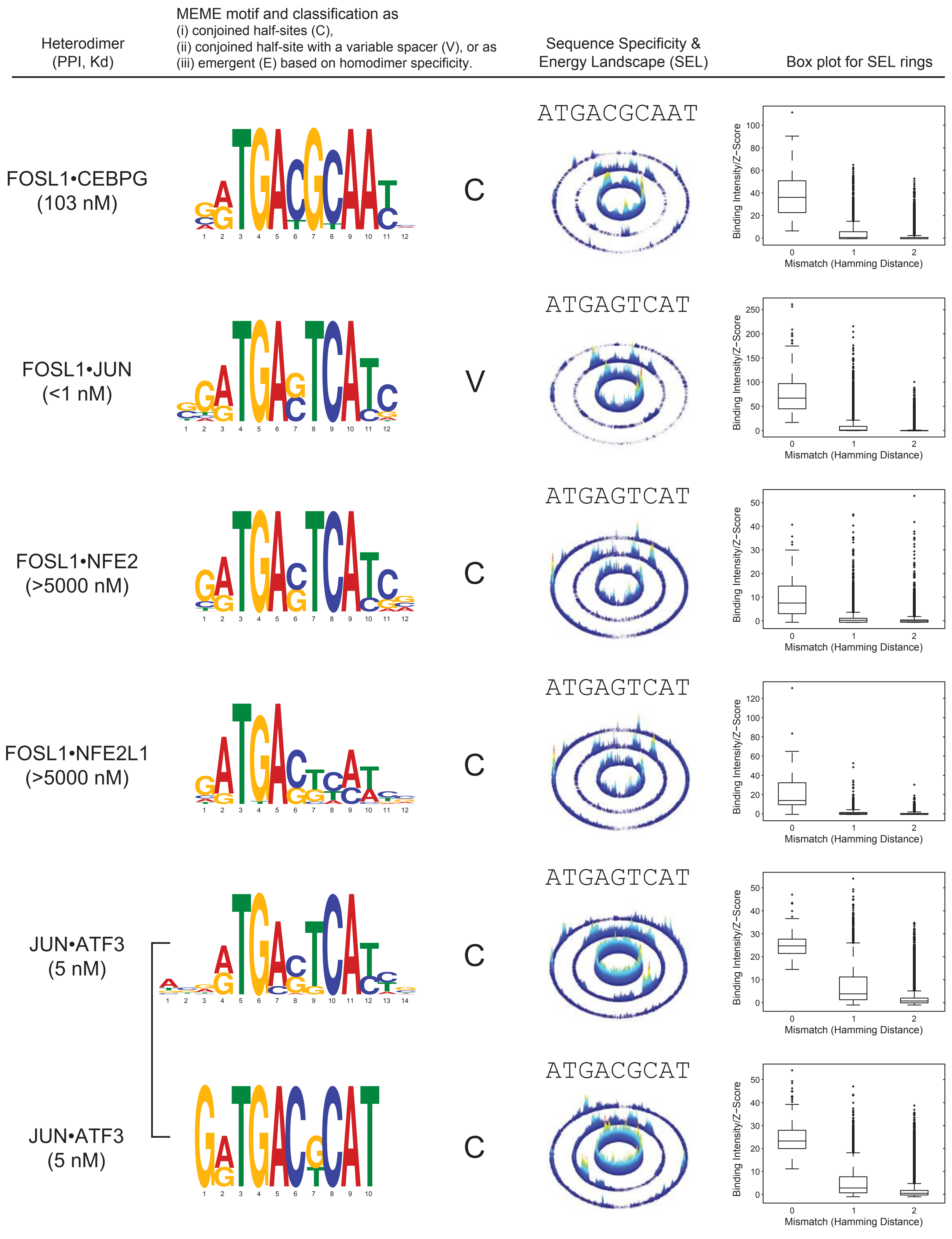

Heterodimer (PPI, Kd)
MEME motif and classification as
(i) conjoined half-sites (C),
(ii) conjoined half-site with a variable spacer (V), or as
(iii) emergent (E) based on homodimer specificity.
Sequence Specificity & Energy Landscape (SEL)
Box plot for SEL rings
FOSL1•CEBPG (103 nM)
C
ATGACGCAAT
FOSL1•JUN (<1 nM)
V
ATGAGTCAT
FOSL1•NFE2 (>5000 nM)
C
ATGAGTCAT
FOSL1•NFE2L1 (>5000 nM)
C
ATGAGTCAT
JUN•ATF3 (5 nM)
C
ATGAGTCAT
JUN•ATF3 (5 nM)
C
ATGACGCAT
Binding Intensity/Z-Score
Mismatch (Hamming Distance)

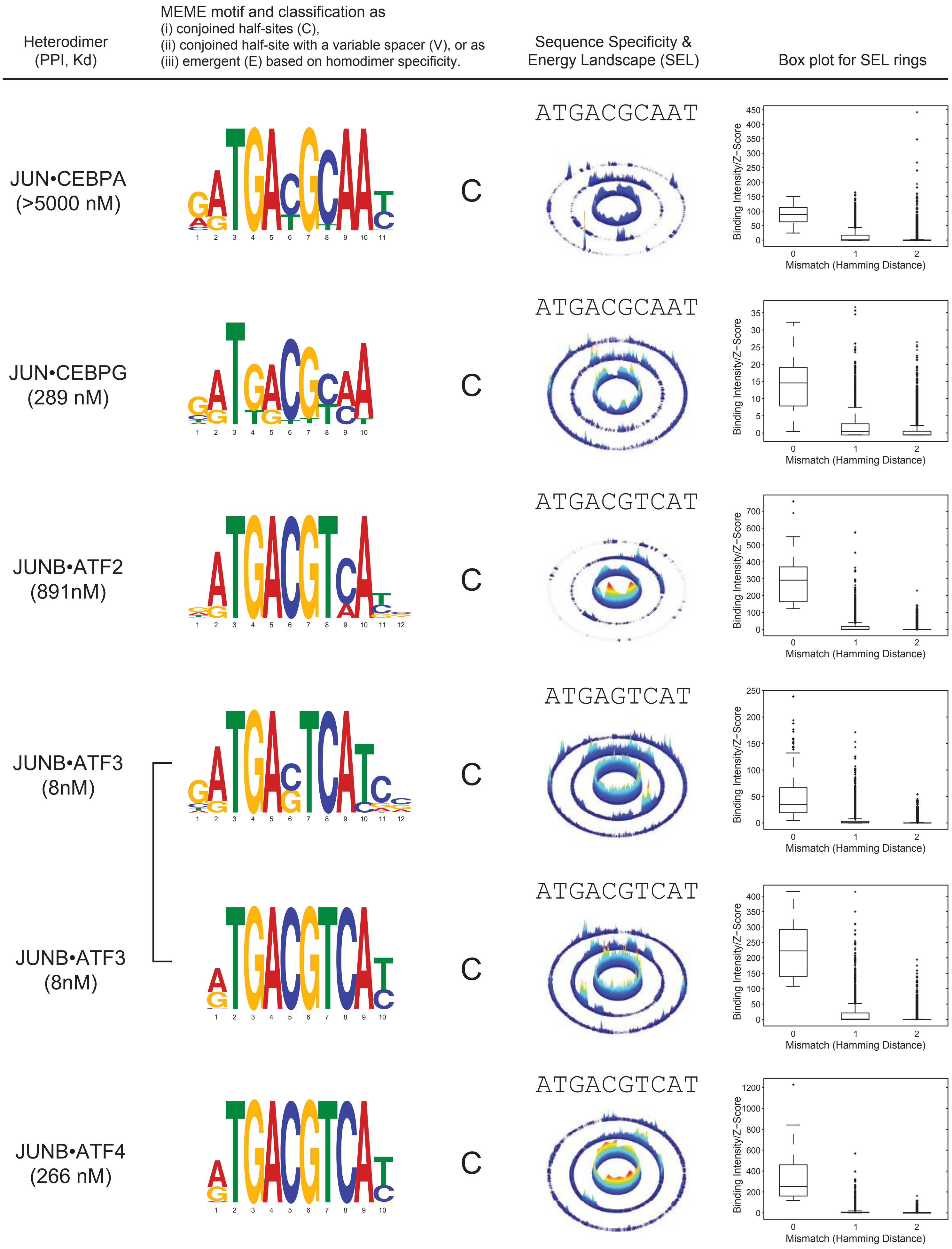
Heterodimer
(PPI, Kd)
MEME motif and classification as
(i) conjoined half-sites (C),
(ii) conjoined half-site with a variable spacer (V), or as
(iii) emergent (E) based on homodimer specificity.
Sequence Specificity &
Energy Landscape (SEL)
Box plot for SEL rings
JUN•CEBPA
(>5000 nM)
C
ATGACGCAAT
JUN•CEBPG
(289 nM)
C
ATGACGCAAT
JUNB•ATF2
(891nM)
C
ATGACGTCAT
JUNB•ATF3
(8nM)
C
ATGAGTCAT
JUNB•ATF3
(8nM)
C
ATGACGTCAT
JUNB•ATF4
(266 nM)
C
ATGACGTCAT
Binding Intensity/Z-Score
Mismatch (Hamming Distance)

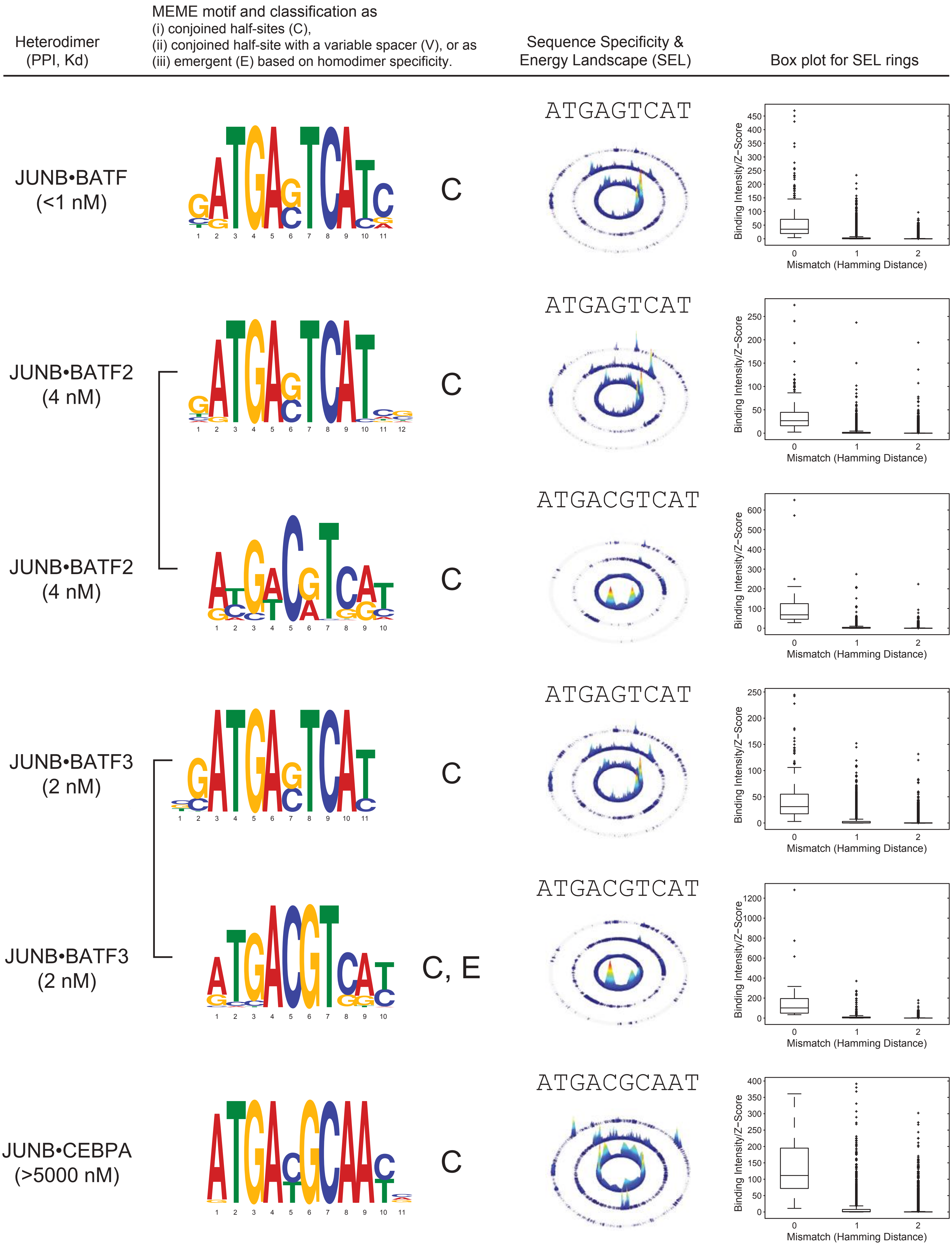
Heterodimer (PPI, Kd)
MEME motif and classification as
(i) conjoined half-sites (C),
(ii) conjoined half-site with a variable spacer (V), or as
(iii) emergent (E) based on homodimer specificity.
Sequence Specificity & Energy Landscape (SEL)
Box plot for SEL rings
JUNB•BATF (<1 nM)
C
ATGAGTCAT
JUNB•BATF2 (4 nM)
C
ATGAGTCAT
JUNB•BATF2 (4 nM)
C
ATGACGTCAT
JUNB•BATF3 (2 nM)
C
ATGAGTCAT
JUNB•BATF3 (2 nM)
C, E
ATGACGTCAT
JUNB•CEBPA (>5000 nM)
C
ATGACGCAAT
Binding Intensity/Z-Score
Mismatch (Hamming Distance)

Heterodimer (PPI, Kd)	MEME motif and classification as (i) conjoined half-sites (C), (ii) conjoined half-site with a variable spacer (V), or as (iii) emergent (E) based on homodimer specificity.		Sequence Specificity & Energy Landscape (SEL)	Box plot for SEL rings
JUNB•CEBPE (1697 nM)	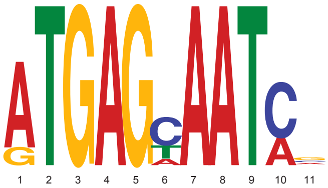	V	ATGACGCAAT 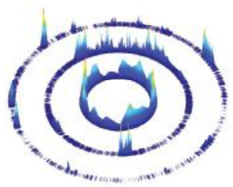	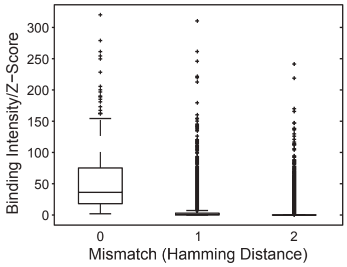
JUNB•CEBPG (>5000 nM)	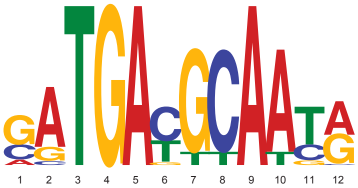	C	ATGACGCAAT 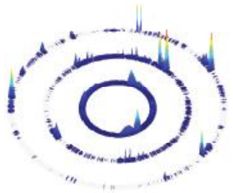	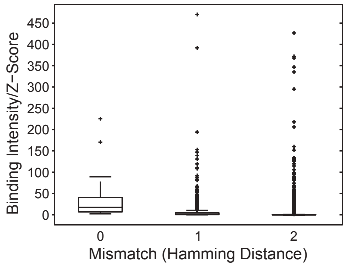
JUNB•CREBZF (>5000 nM)	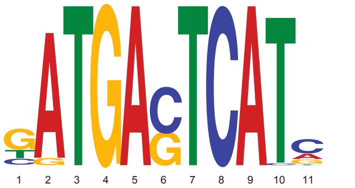	C	ATGAGTCAT 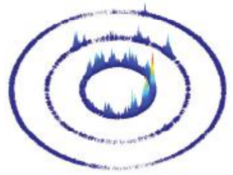	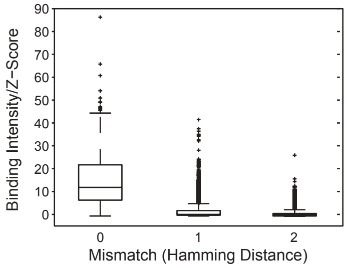
JUNB•DBP (>5000 nM)	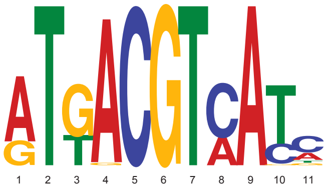	C	ATGACGTCAT 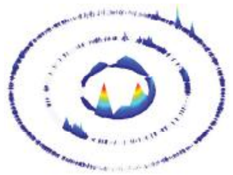	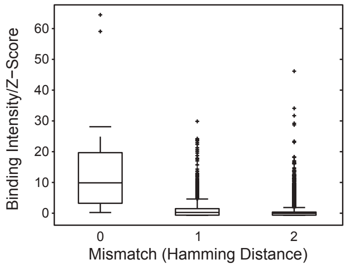
JUNB•FOS (2 nM)	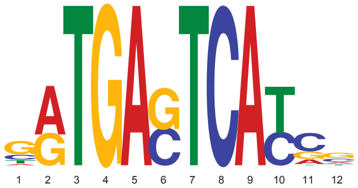	C	ATGAGTCAT 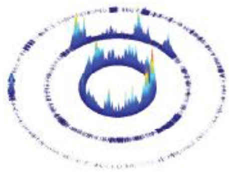	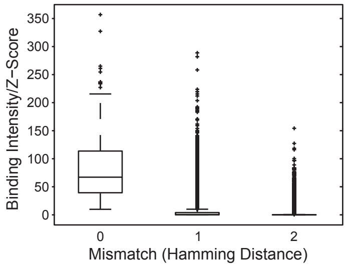
JUNB•FOSL1 (5 nM)	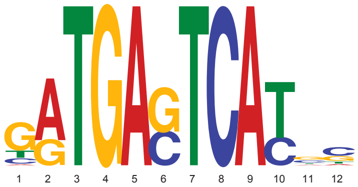	C	ATGAGTCAT 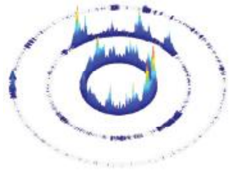	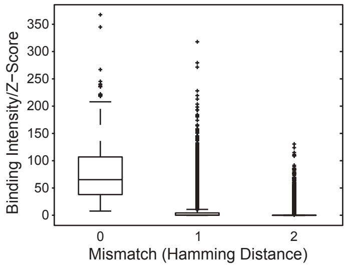

Heterodimer (PPI, Kd)	MEME motif and classification as (i) conjoined half-sites (C), (ii) conjoined half-site with a variable spacer (V), or as (iii) emergent (E) based on homodimer specificity.		Sequence Specificity & Energy Landscape (SEL)	Box plot for SEL rings
JUNB•HLF (>5000 nM)	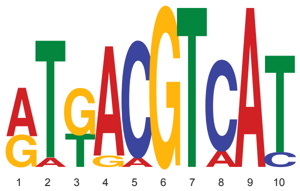	C	ATGACGTCAT 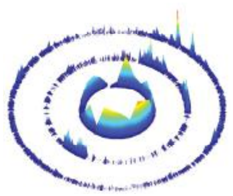	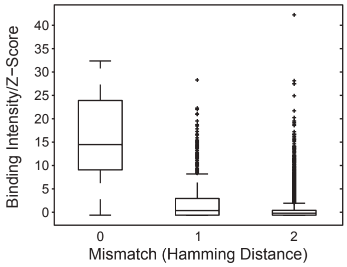
JUNB•JUN (>5000 nM)	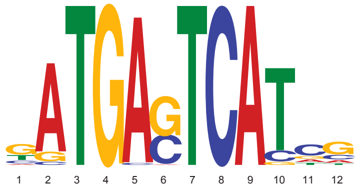	C	ATGAGTCAT 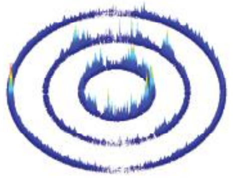	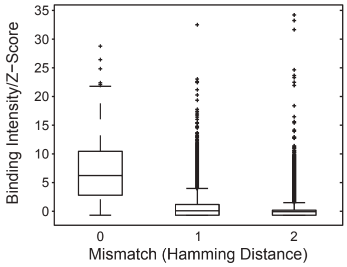
JUNB•JUN (>5000 nM)	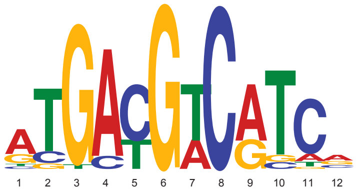	C	ATGACGTCAT 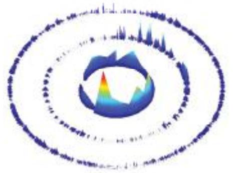	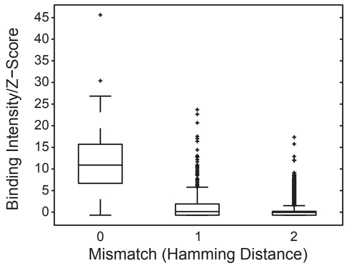
JUNB•NFE2 (>5000 nM)	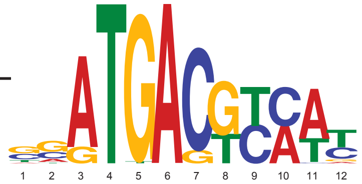	C	ATGACGTCAT 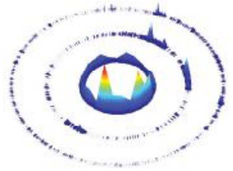	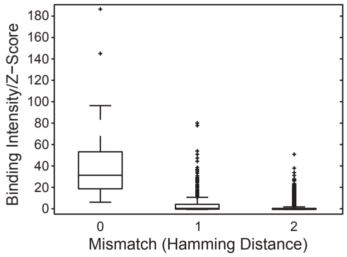
JUNB•NFE2 (>5000 nM)			ATGAGTCAT 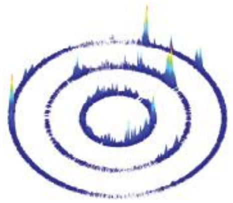	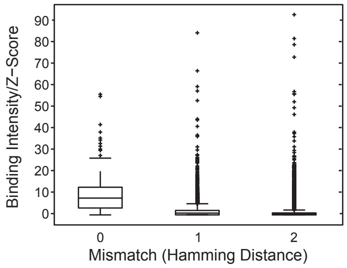

Heterodimer (PPI, Kd)	MEME motif and classification as (i) conjoined half-sites (C), (ii) conjoined half-site with a variable spacer (V), or as (iii) emergent (E) based on homodimer specificity.		Sequence Specificity & Energy Landscape (SEL)	Box plot for SEL rings
JUNB•NFE2L1 (>5000 nM)	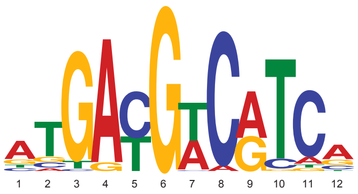	C	ATGACGTCAT 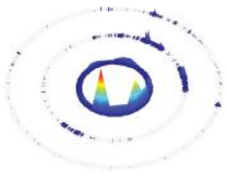	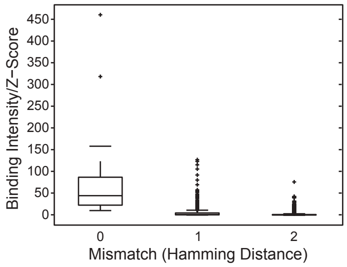
JUNB•NFE2L1 (>5000 nM)	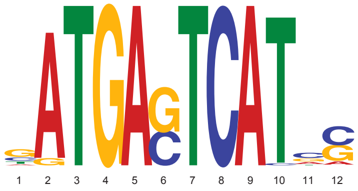	C	ATGAGTCAT 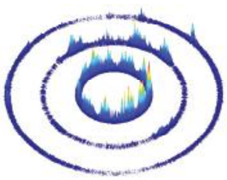	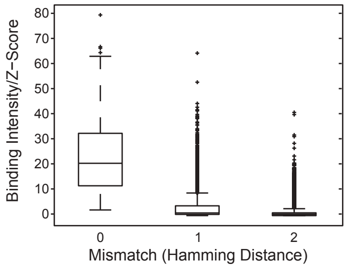
MAFB•ATF4 (193 nM)	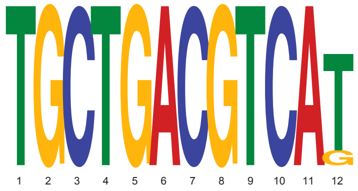	C	TGCTGACGTCAT 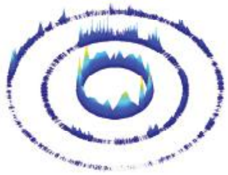	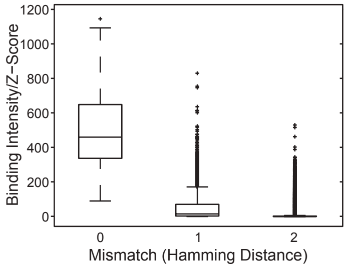
MAFB•ATF5 (>5000 nM)	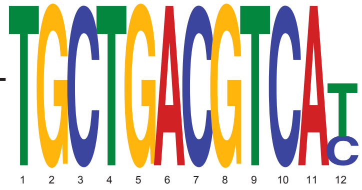	C	TGCTGACGTCAT 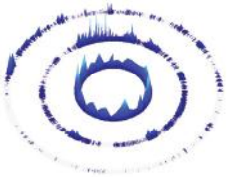	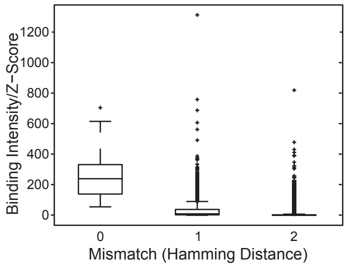
MAFB•ATF5 (>5000 nM)	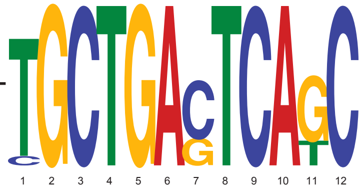	C	TGCTGAGTCAT 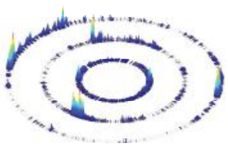	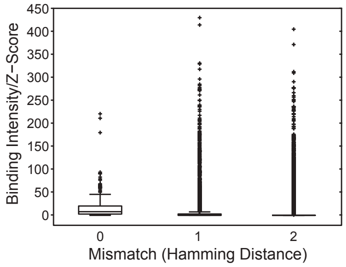

Heterodimer (PPI, Kd)	MEME motif and classification as (i) conjoined half-sites (C), (ii) conjoined half-site with a variable spacer (V), or as (iii) emergent (E) based on homodimer specificity.		Sequence Specificity & Energy Landscape (SEL)	Box plot for SEL rings
MAFB•FOSL1 (152 nM)	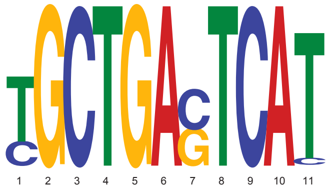	C	TGCTGAGTCAT 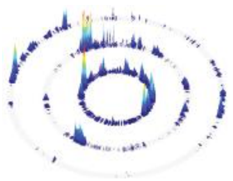	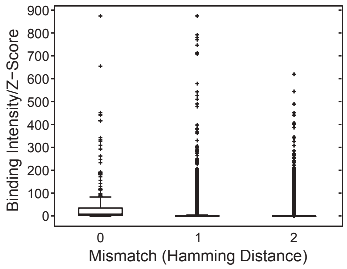
NFE2•MAFG (1340 nM)	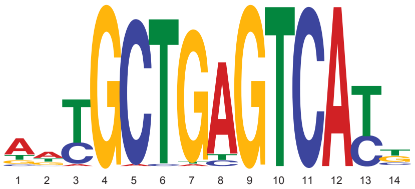	C	TGCTGAGTCAT 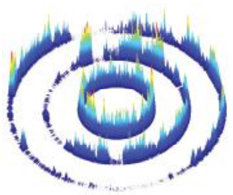	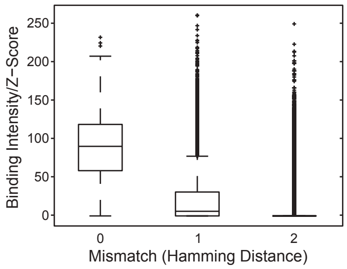
NFE2L1•MAFG (<1 nM)	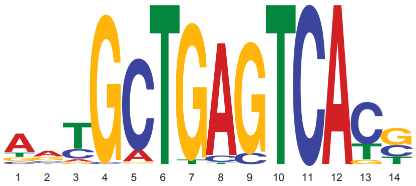	C	TGCTGAGTCAT 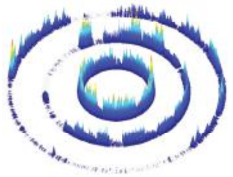	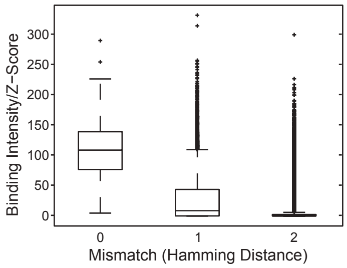
